# Human-assisted sound event recognition for home service robots

**DOI:** 10.1186/s40638-016-0042-2

**Published:** 2016-06-02

**Authors:** Ha Manh Do, Weihua Sheng, Meiqin Liu

**Affiliations:** School of Electrical and Computer Engineering, Oklahoma State University, Stillwater, OK 74078 USA; Beijing Advanced Innovation Center for Imaging Technology, Capital Normal University, Beijing, 100048 China; College of Electrical Engineering, Zhejiang University, Hangzhou, 310027 China

**Keywords:** Sound event recognition, Home service robot, Human–robot collaboration, Auditory perception, Elderly care

## Abstract

This paper proposes and implements an open framework of active auditory learning for a home service robot to serve the elderly living alone at home. The framework was developed to realize the various auditory perception capabilities while enabling a remote human operator to involve in the sound event recognition process for elderly care. The home service robot is able to estimate the sound source position and collaborate with the human operator in sound event recognition while protecting the privacy of the elderly. Our experimental results validated the proposed framework and evaluated auditory perception capabilities and human–robot collaboration in sound event recognition.

## Background

For the elderly who live independently in their own residence, home service robots can play as a social companion to collaborate and interact with the elderly. One important communication channel in human daily life is the sound, which includes voice and non-voice. Therefore, it is desirable to equip home service robots with sound processing capability. The robot needs to know where the sound sources are located even when multiple sound sources exist. This can help the robots respond to human commands and events more accurately. Furthermore, it is very important for the home service robots to understand the sound events that are generated by human’s daily activities such as cooking, drinking, washing hands, having shower, using a toilet, sounds associated with anomalous behaviours such as falling on the floor. Sound event recognition helps the robot not only monitor elderly’s activities but also detect anomalies happening in their home. Such a human-aware capability frees the robot to do its daily routine work, while being able to take care of the elderly more proactively and effectively.

In recent years, home service robots for the elderly living alone at home have been receiving growing interest. There are already some commercial assistive social robots for elderly care, such as Pearl, Aibo [1], Care-o-Bots [2], Homie, iCat, Paro, and Huggable. [3]. Some of them, for example Pearl and Care-o-Bots, can recognize words, synthesize speech, work as autonomous guidance or telepresence robots, and remind people about daily activities such as eating, drinking, and taking medicine, but they do not have the auditory capability that enables the robots to understand both voice and event sounds. Several research and development robots for domestic environments, such as Johnny [4] and European CompanionAble project’s Hector [5], were equipped with mapping, navigation, friendly graphical user interface (GUI), speech recognition, etc. The PR2 robot platform was programmed to help a severely disabled man [6]. However, these robots are not able to recognize sound events, especially in a multi-source environment.

Recently, sound event recognition (SER) has received growing attention from the research community. Various approaches have been developed for SER. Most approaches are derived from the research on speech recognition, such as hidden Markov models (HMM) with mel-frequency cepstral coefficients (MFCCs) [7], Gaussian mixture models (GMMs) with LFCC [8], and iGMM [9]. On the other hand, nonparametric learning methods have been proposed, such as the technique based on sparse coding of stabilized auditory images (SAIs) [10]. Recently, principal component analysis and linear discriminant analysis are applied to the scale-frequency map to generate the feature for sound event classification based on the multi-class SVMs [11]. SVM-based methods show high performance on sound event recognition. MFCC-SVM can achieve an accuracy rate of 74.50 % [12]. Several works have applied deep neural networks (DNNs) for polyphonic sound event recognition, such as multi-label DNNs [13], novel spiking neural network system [14], and DNN-based framework with the different spectrogram image-based front features such as Google-style SAI features and spectrogram image features (SIFs) [15]. These works were mainly tested on the sound event databases that are the mixtures of sounds in both indoor and outdoor environments. However, only a few sound events that are associated with the daily activities of the elderly in home environment have been evaluated by the auditory systems on the robots.

Humans have strong capability of auditory perception, which enables them to not only understand the voice and non-voice sounds, but also sort through the incoming information. It is highly desired to equip the robots with both speech recognition and sound event recognition capability. Speech recognition has been well researched, and there is even open-source software available, such as PocketSphinx [16] and Julius [17]. However, sound event recognition is still challenging due to the diversity of the sounds associated with the same event. For example, even the same event of an elderly person falling on the floor can create different sounds, depending on where the fall occurs. Different events also produce different sounds, which makes it extremely hard to preprogram the robot with a small set of training data. Moreover, it is not easy for humans to recognize what is going on from hearing domestic audio without context. The knowledge of the context is one important factor that allows the humans to hear in unconstrained environments and helps them form predictions and guide their perception of the environment [18]. These examples tell us that such a robot should gradually learn the audio events in its unique environment and, whenever possible, get assistance from humans who can provide guidance on the auditory learning process. It is also desired that the robot can provide contextual information for the humans by estimating the position of sound sources.

In this paper, we propose that by putting a human in the loop of sound event recognition, a robot can better understand and more quickly adapt to its environment. The human-assisted sound event recognition for home service robots is proposed and implemented based on our previous work [19]. Using a microphone array, the robot is able to localize and separate multiple sound sources. Then, the robot classifies the separated sounds into voice and non-voice. The non-voice sounds along with location data can be sent to a human caregiver for recognition and labelling. Since only non-voice sound is sent to outside, the privacy of the elderly can be protected. With more and more labelled event sound data, the robot can train its sound event recognition algorithm to achieve better accuracy, therefore enabling incremental auditory learning. Such human–robot collaboration in sound event recognition allows developing social intelligence through active auditory learning.

This paper is organized as follows: The next section describes the design of the robot platform with an auditory system. Then, we describe the implementation of auditory services and human-assisted sound event recognition, respectively. Following that, experiments and results are presented to verify the working of the framework. Finally, we conclude the paper and indicate the potential future work.

**Fig. 1:**
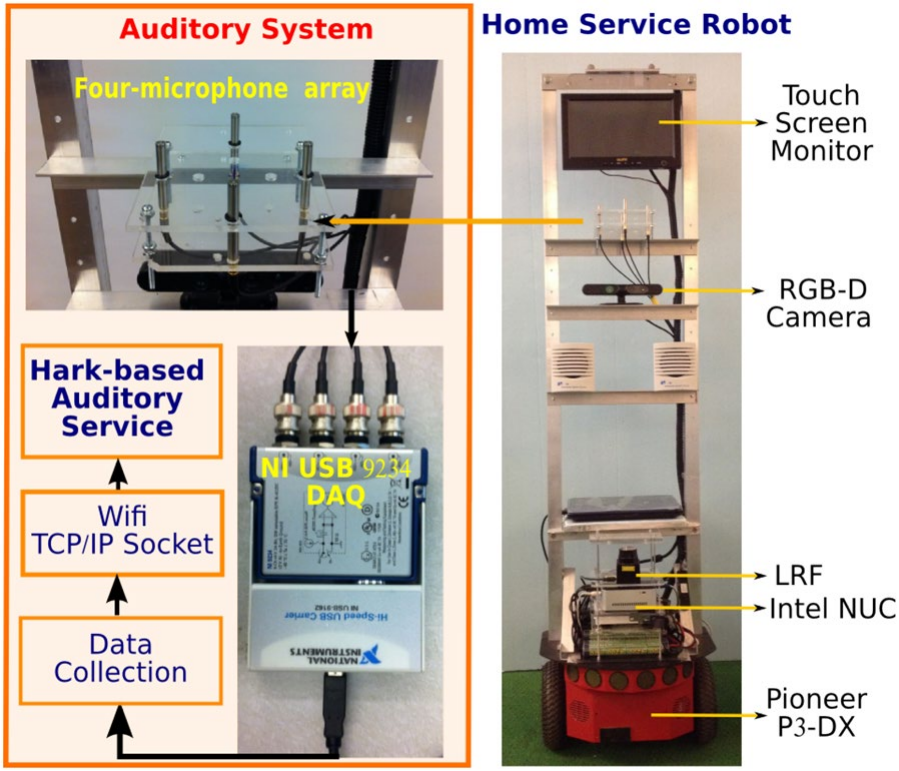
The home service robot platform and the auditory system

**Fig. 2:**
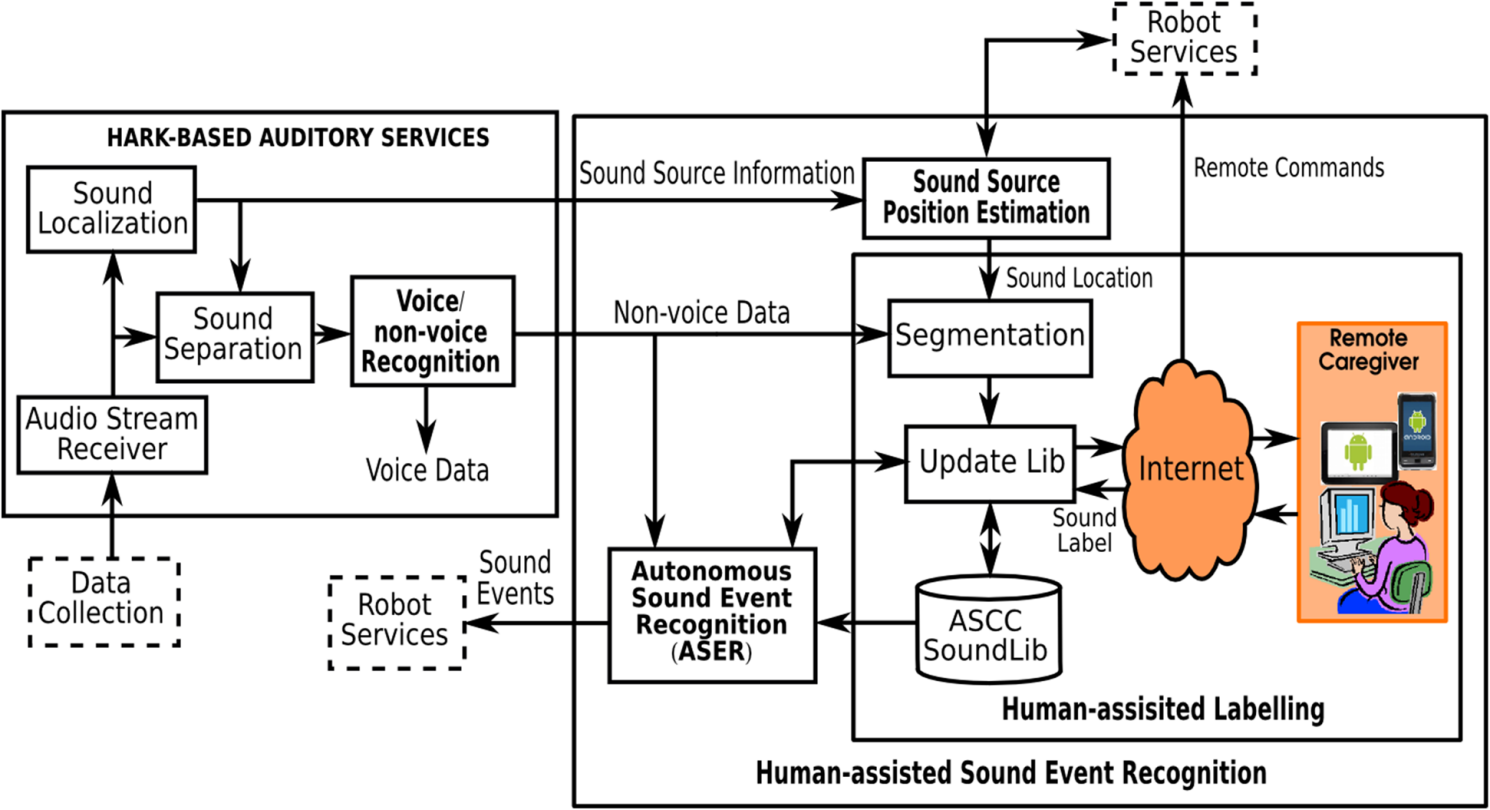
The framework of human-assisted sound event recognition

## Robot platform with an auditory system

The ASCC home service robot is built with a pre-existing mobile platform as shown in Fig. 1. Besides basic features such as simultaneous localization and mapping (SLAM) and autonomous navigation based on 2D maps, the robot also has the capability of auditory perception to collaborate with the remote caregiver to recognize sound events.

### Home service robot platform

#### Robot hardware

Our home service robot as shown in Fig. 1 was built on a Pioneer P3-DX base with approximately a 1.5-m-long aluminium frame holding up a touch screen monitor, which is used for video communication and graphic user interface. Mounted on it are a RGB and Depth (RGB-D) camera, a laser range finder (LRF), an Intel NUC minicomputer, and a netbook computer. The RGB-D camera mounted on top of the robot is an ASUS Xtion PRO LIVE, which allows developing functions such as 3D mapping, obstacle avoidance, and gesture-based control. The LRF, a Hokuyo URG-04LX-UG01, is a low-power LRF with a wide range up to 5600 mm × 240° and an accuracy of 30 mm.

#### Robot software

The software for the robot was developed on ROS [20] which runs in Ubuntu on the Intel NUC minicomputer. For the basic functions in the robot, we utilized existing packages from the ROS repositories to set up drivers that interface with the robot base, the Hokuyo LRF, and the Xtion camera. Two main services including SLAM and navigation were developed based on existing ROS packages. SLAM was based on Rao-Blackwellized particle filters [21]. Motion planning and autonomous navigation were based on the particle filter-based localization method and the adaptive (or KLD-sampling) Monte Carlo localization approach [22].

### Auditory system

#### Auditory hardware

The hardware for auditory perception as shown in Fig. 1 was built with 4 G.R.A.S IEPE (integrated electronic piezoelectric) microphones and an NI USB-9234 DAQ (data acquisition). This set of microphones has high sensitivity at 50 mV/Pa, a wide frequency range up to 20 kHz, and a large dynamic range topping at around 135 dB. The DAQ is a USB-based four-channel C Series dynamic signal acquisition module for high-accuracy audio-frequency measurements from IEPE and non-IEPE sensors. It can deliver a dynamic range of 102 dB, incorporate programmable AC/DC coupling and IEPE signal conditioning for accelerometers and microphones, as well as digitize signals at rates up to 51.2 kHz per channel with built-in antialiasing filters that automatically adjust to the sampling rate.

#### Auditory software

The auditory software platform was based on HARK [23], which is an open-sourced audition software consisting of modules for acoustic signal processing, sound localization and separation, speech recognition, and audio streaming. The data collection program was developed to capture the audio data from the microphones, filter them out, and send them to AudioStreamFromMic block (an audio stream receiver) through a WiFi TCP/IP socket for further processing.

## HARK-based auditory services

As shown in Fig. 2, developed using HARK, audition services perform sound localization, sound separation, and voice/non-voice recognition from the four-channel audio stream coming from the data collection module.

### Sound localization and separation

Sound localization is implemented based on the GEVD (generalized eigenvalue decomposition) method [24]. Direction of arrival (DOA) in the horizontal plane is estimated by the multiple signal classification (MUSIC) method [25], which has shown the best performance. This method localizes sound sources based on source positions and impulse responses (transfer function) of microphones. The transfer function generally varies depending on the shape of the room and the relative positions between microphones and sound sources [26]. However, when ignoring acoustic reflection and diffraction, and given that the relative position of microphones and sound sources is known, the transfer function $H_{D}(k_{i})$ is limited only to the sound source direction and calculated by the following Equation [26]:

1 $$\begin{aligned} H_{Dm,n}(k_{i})=exp\left( \frac{-j2\pi \omega _{i}}{c}r_{m,n}\right) \end{aligned}$$

where *c* is the speed of sound; $\omega _{i}$ is the frequency in the frequency bin $k_{i}$; $r_{m,n}$ is the difference between the distance from the microphone *m* to the sound source *n* and the distance from the reference point of the coordinate system to the sound source *n*.

The sound that is emitted from *N* sound sources is affected by the transfer function $H(k_{i})$ in space and observed through M microphones as expressed by Eq. (2).

2 $$\begin{aligned} X(k_{i})=H(k_{i})S(k_{i}) + N(k_{i}) \end{aligned}$$

where $S(k_{i})$ is the sound source complex spectrum corresponding to the frequency bin $k_{i}$; $N(k_{i})$ is the additive noise that gets into each microphone.

The matrix of a complex spectrum of separated sound $Y(k_{i})$ is obtained from the following equation:

3 $$\begin{aligned} Y(k_{i})=W(k_{i})X(k_{i}) \end{aligned}$$

The separation matrix $W(k_{i})$ is estimated by Geometric-Constrained High-order Source Separation (GHDSS) [27], which has the highest total performance in various acoustic environments. With the source direction from sound localization, the separated sound $Y(k_{i})$ is likely close to its sound source $S(k_{i})$.

### Voice/non-voice recognition

**Fig. 3:**
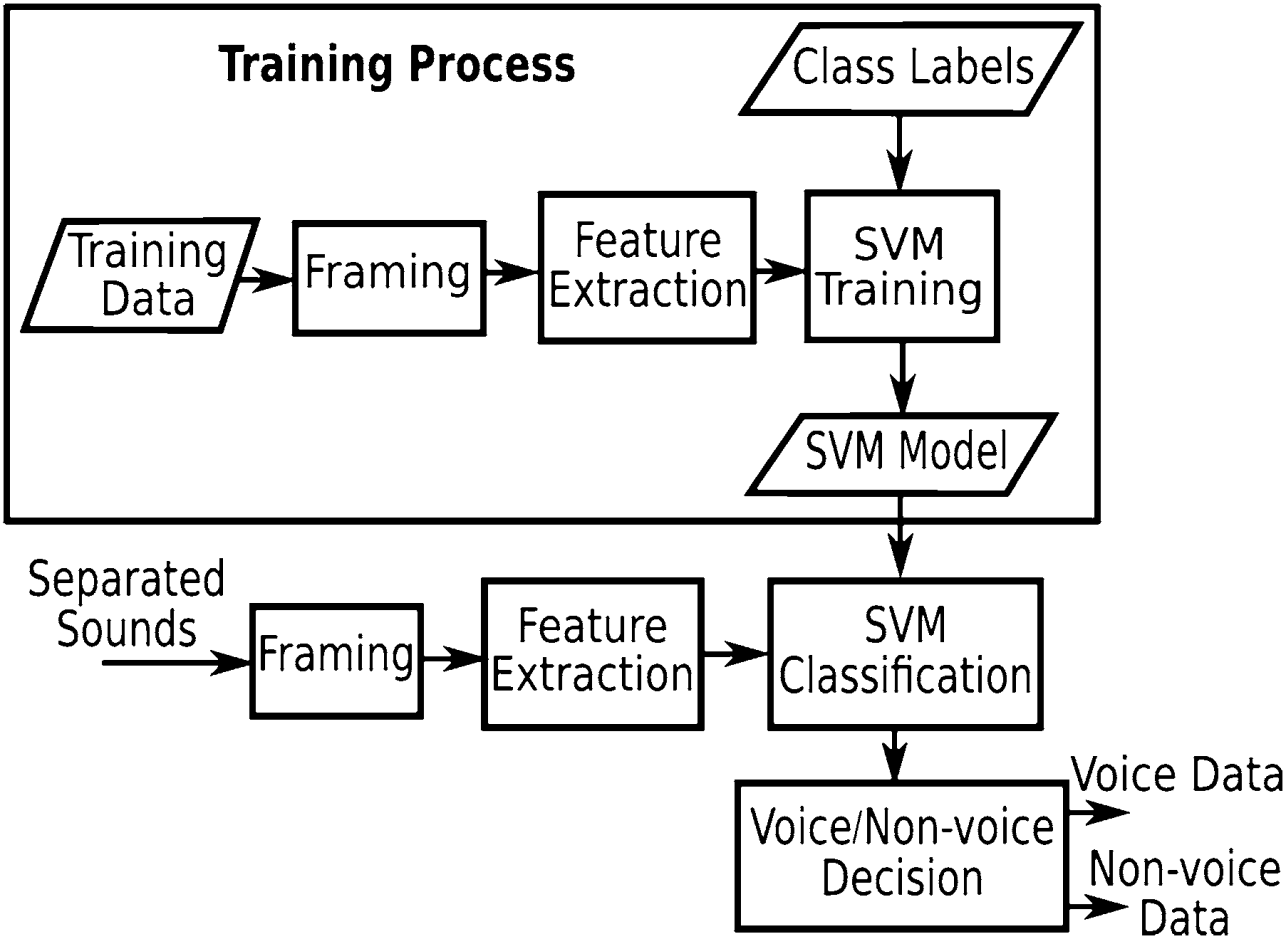
Voice/non-voice recognition

The separated sounds are classified into voice and non-voice. To achieve this, we use the support vector machine (SVM) algorithm. In SVM, the kernel function is applied to transform nonlinear and high-dimensional feature vectors into simpler feature vectors that can be classified by the optimal decision hyperplane using linear discriminant functions. The kernel function widely used in SVM for audio applications is the Gaussian radial basis function (RBF) as follows:

4 $$\begin{aligned} K(x_{i},x_{j})=exp\left( -\gamma \Vert x_{i}-x_{j}\Vert ^{2}\right) \end{aligned}$$

where $\gamma$ is a control parameter estimated from the variance of the distribution function of the training data. RBF-SVM aims to construct the decision function for the data point *x* based on N support vectors $\{x_{k}\}_{k=1}^{N}$ and labels $\{y_{k}\}_{k=1}^{N}$ as follows:

5 $$\begin{aligned} y(x)=sign\left[ \sum _{k=1}^{N}\alpha _{k}y_{k}K(x_{k},x)+b\right] \end{aligned}$$

where $\alpha _{k}$ is the weight assigned to the support vector $x_{k}$, b is a constant bias. As shown in Fig. 3, the RBF-SVM was implemented for voice/non-voice recognition based on Voice Active Detection proposed in [28]. In order to train the SVM, the audio training data consisting of labelled voice and non-voice segments are decomposed into frames. The 36-MFCC feature vectors are computed for each frame. The trained SVM model can classify frames of separated sounds into voice or non-voice.

## Human-assisted sound event recognition

To the best of our knowledge, this is the first work that proposed a framework for human–robot collaboration in sound event recognition. The framework allows the robot not only to capture and separate acoustic events but also to estimate the context of sound events and send the audio data along with their contextual information to human caregivers for labelling. Human-assisted sound event recognition contains three functions: sound source position estimation, human-assisted labelling, and autonomous sound event recognition as shown in Fig. 2. The robot is able to estimate the sound source position and send only non-voice sounds along with location data to a human caregiver for recognition and labelling. The labelled data are stored in the sound library (SoundLib), which can be used to train the sound event recognition algorithm on the robot.

**Fig. 4:**
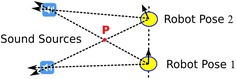
Sound source position estimated by triangulation

### Sound source position estimation

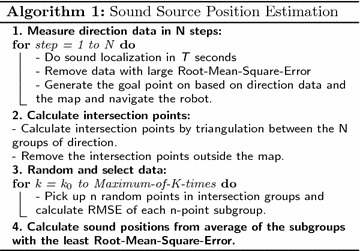



The direction of the sound source can be estimated using the sound localization method described above. With one stationary microphone array, it is hard to estimate the sound source position. However, the home service robot can move around, which makes it possible to use triangulation to localize the sound source. Figure 4 shows an example of using triangulation to estimate the positions of two sound sources. If the robot can measure the sound direction at two different positions on the 2D map, the sound position can be estimated by calculating the intersection of two lines pointing to the sound sources from the robot positions. This method may create a undesired intersection point like point P as shown in Fig. 4. However, this point moves when the robot measures at another position. Therefore, it can be eliminated given the assumption that the sound sources are stationary. With multiple steps, the robot can improve the accuracy of position estimation using the RANdom SAmple Consensus (RANSAC) algorithm [29]. The sound source position estimation algorithm is shown in Algorithm 1.

### Human-assisted labelling

Many non-voice sounds are generated by human activities at home, such as having shower, flushing a toilet, soaping hands, washing hands, brushing teeth in the bathroom; using a microwave oven, and boiling water in the kitchen. Recognizing these sounds can help the robot understand human activities. However, due to the lack of sufficient training samples in the individual home environment, it is very hard to achieve satisfactory non-voice sound recognition. Therefore, as shown in Fig. 2, we propose to let the robot and the human caregiver collaborate to recognize it. Basically, the robot sends the segment of non-voice sound to the caregiver, who then recognizes it and labels it through a user interface. Such an interface can be on a computer, or a mobile device such as a tablet or smartphone. The sound library (SoundLib) consists of labelled sound events, which can be used in the training of the sound event recognition algorithm, therefore enabling incremental learning. When sufficient labelled data are available, the robot will be able to use the recognition algorithm to accurately recognize the event sounds.

**Fig. 5:**
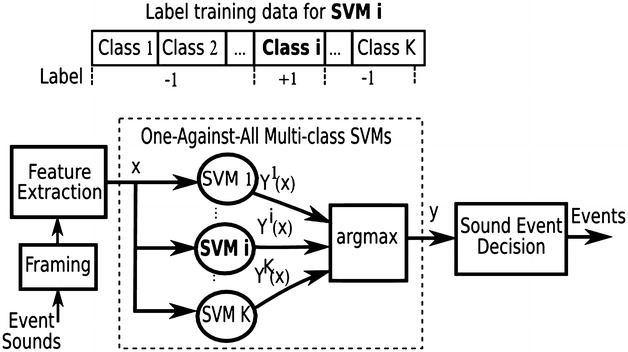
SVM-based sound event recognition

**Fig. 6:**
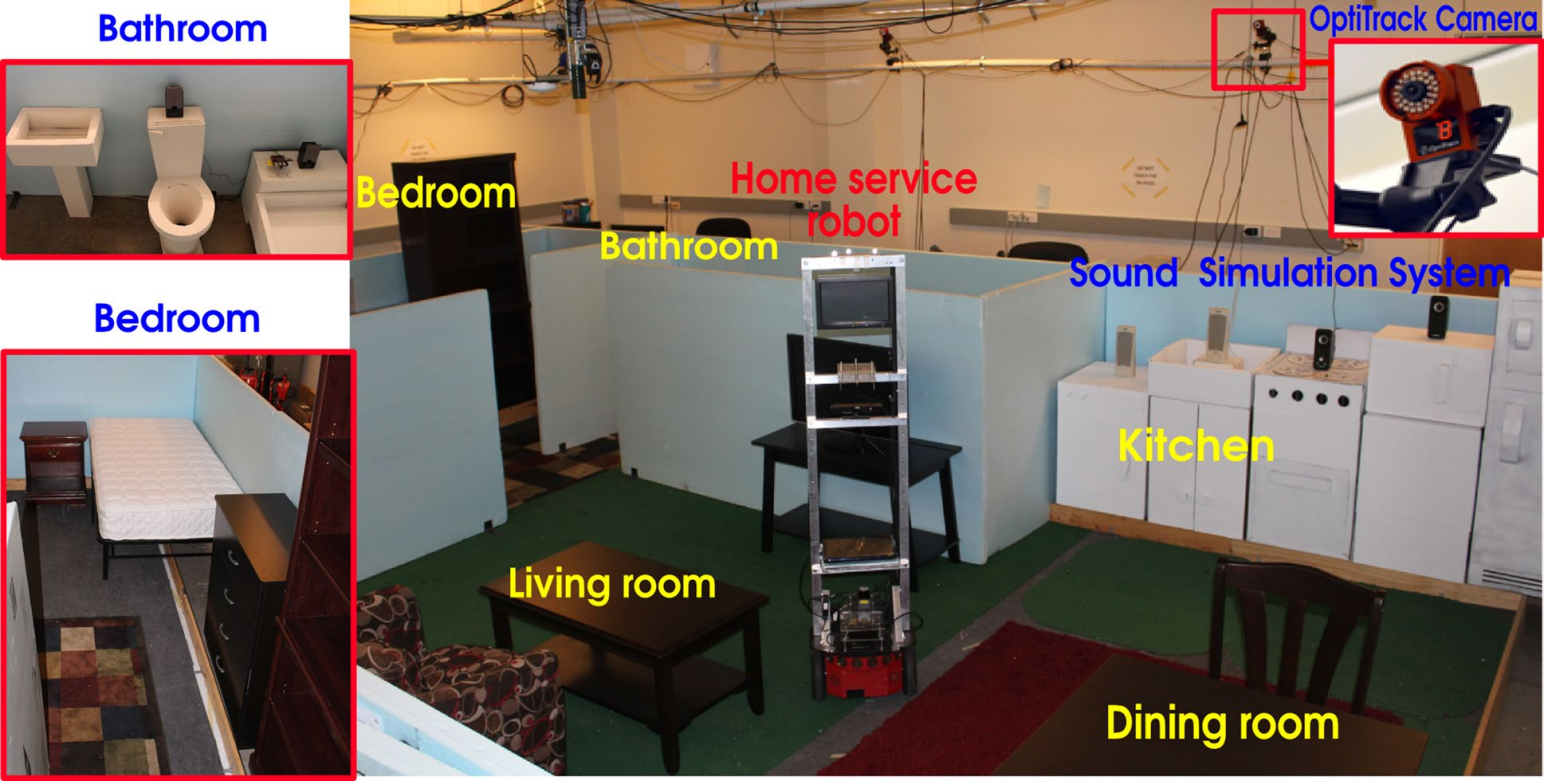
Smart home testbed

**Fig. 7:**
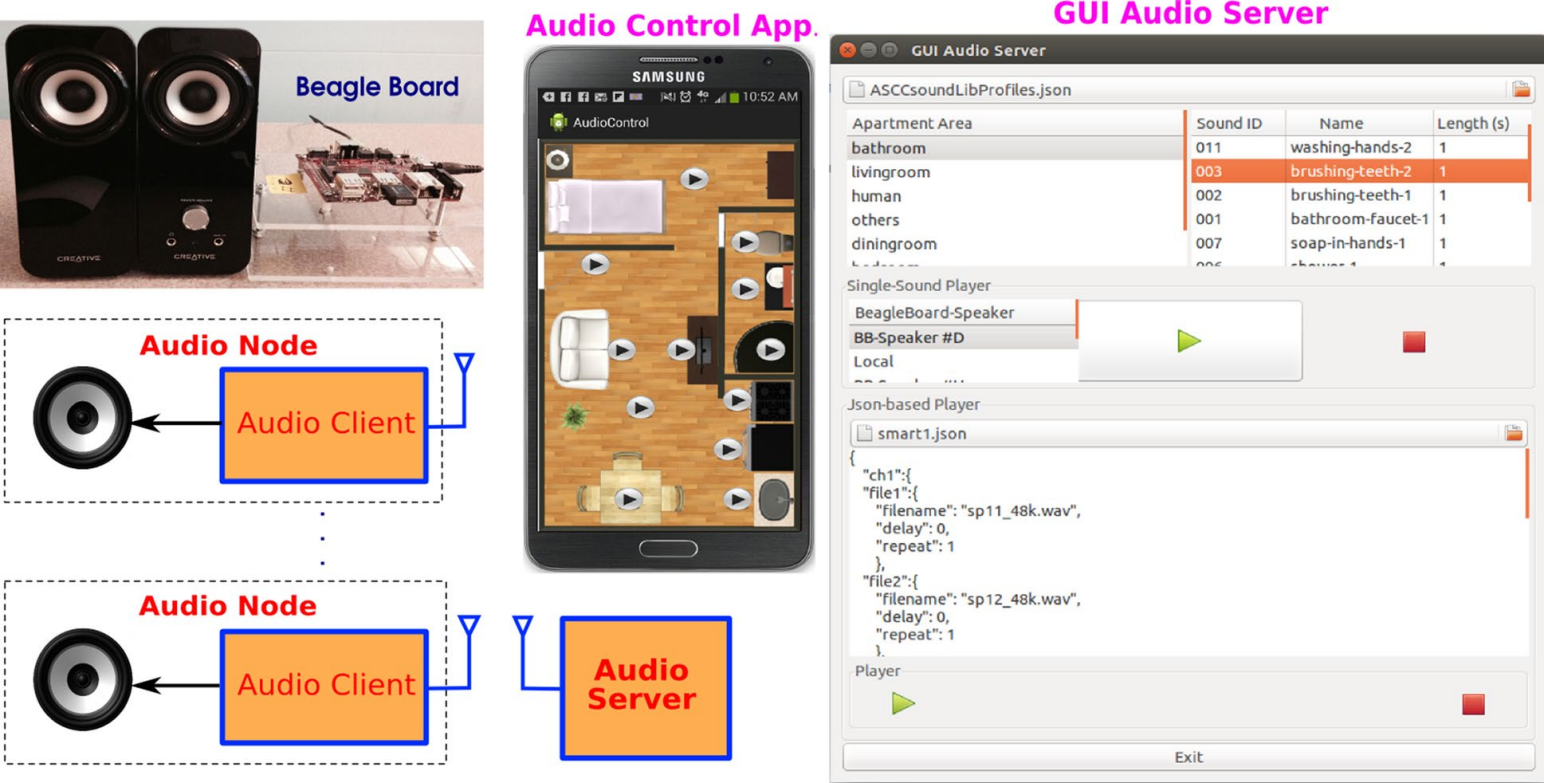
Sound simulation system

### Autonomous sound event recognition

In this work, we implement sound event recognition based on multiple class SVMs with MFCC features. SVM was originally designed for binary classification. However, it can be utilized to construct multi-class classification or recognition in several approaches. The first approach is decomposition by combining multiple SVMs using One-Against-One (OAO), One-Against-All (OAA), or hierarchical binary tree methods. The second is global or all-together approach by solving a single optimization problem. Compared with other methods by experiments on large problems, Hsu and Lin concluded that the OAA method could be more suitable for practical use [30]. As shown in Fig. 5, the multi-class classification is constructed by K SVM models where K is the number of classes. SVM models are trained by the One-Against-All (OAA) method. The *i*th SVM is trained with all training data in the *i*th class with positive labels and all other training data with negative labels. After the training phase, there are K decision functions as follows:

6 $$\begin{aligned} Y^{i}(x)=\sum _{k=1}^{N^{i}}\alpha ^{i}_{k}y^{i}_{k}K \left( x^{i}_{k}, x\right) +b^{i},\quad i=1,\ldots , K \end{aligned}$$

where $\alpha ^{i}_{k}$ is the weight assigned to the support vector $x^{i}_{k}$; $b^{i}$ is a constant bias of the *i*th SVM.

The input *x* is classified into the class which has the largest value of the decision functions:

7 $$\begin{aligned} y = {\mathrm{arg\,max}}_{i=1,\ldots ,k} \left\{ Y^i(x) \right\} \end{aligned}$$

SVM-based SER is implemented by using OAA, RBF, and 36-MFCC feature vectors.

## Experiments and results

We conducted physical experiments to test and evaluate our framework. A smart home testbed is set up in our laboratory at the area of 16 ft $\times$ 22 ft as shown in Fig. 6. It simulates a small apartment, which includes a living room, a bedroom, a kitchen, a dinning room, and a bathroom. Furniture is set up in different rooms. During the experiment, we use the OptiTrack system [31] to provide the location ground truth of the robot, the speakers, and the human to evaluate sound localization as well as sound source position estimation. We developed a system to simulate the multiple sound events like those in a typical house. As shown in Fig. 7, the sound simulation system includes multiple audio nodes, an audio server, and an audio control application. The audio nodes were developed using minicomputers (Beagleboards) and speakers. The sound events in the bathroom, kitchen, living room, bedroom, and dinning room were recorded or collected from the Internet. Currently, our sound library (ASCC SoundLib) has 50 sound event files and 50 speech files. As shown in Fig. 7, audio control programs for the audio server and the android smartphone were developed to play sounds associated with the human activities or play multiple sound event files at the same time on different speakers placed at different locations. For example, it can play both the TV sound in the living room and the shower sound in the bathroom, or play a sequence of sound events related to the cooking activity in the kitchen. The script or schedule for playing sound events can be written in the JSON (JavaScript Object Notation) format.

### Sound localization

Sound localization was tested using the sound simulation system and the OptiTrack system. To fully evaluate the accuracy of the sound localization, the speaker was placed at different directions ($0^{\circ }, \pm 45^{\circ }, \pm 90^{\circ }$, and $\pm 135^{\circ }$) and distances (0.5, 1, 2 and 3 m) with respect to the robot. The OptiTrack system obtains the relative locations between the speaker and the robot, which are treated as the ground truth. For each location, we run the sound localization algorithm 10 times and calculated the mean and the standard deviation, which are given in Table 1. The sound sources can be localized at reasonable accuracy. From Table 1, the detection errors are close in the same distance and not very sensitive to the direction of the sound sources. However, the errors increase with the distance. The standard deviation of errors is $<2^{\circ }$ at 0.5 m and $<4^{\circ }$ at 3 m away from the robot.

**Table 1:** Results of sound localization

Distance	Errors	Direction				
		$0^{\circ }$	$\pm 45^{\circ }$	$\pm 90^{\circ }$	$\pm 135^{\circ }$	Sum
0.5 m	Mean (°)	−0.3	−0.1	−0.2	0.2	−0.2
	Std (°)	1.5	2.0	1.9	1.6	1.7
1 m	Mean (°)	0.6	−0.8	−0.2	0.5	−0.1
	Std (°)	2.2	2.1	2.3	2.0	2.2
2 m	Mean (°)	0.1	0.2	−0.1	0.1	−0.3
	Std (°)	3.1	2.9	3.0	2.7	2.9
3 m	Mean (°)	1.8	0.3	−1.1	−0.9	0.5
	Std (°)	4.2	3.6	4.0	3.7	3.9

### Sound separation and voice/non-voice recognition

**Fig. 8:**
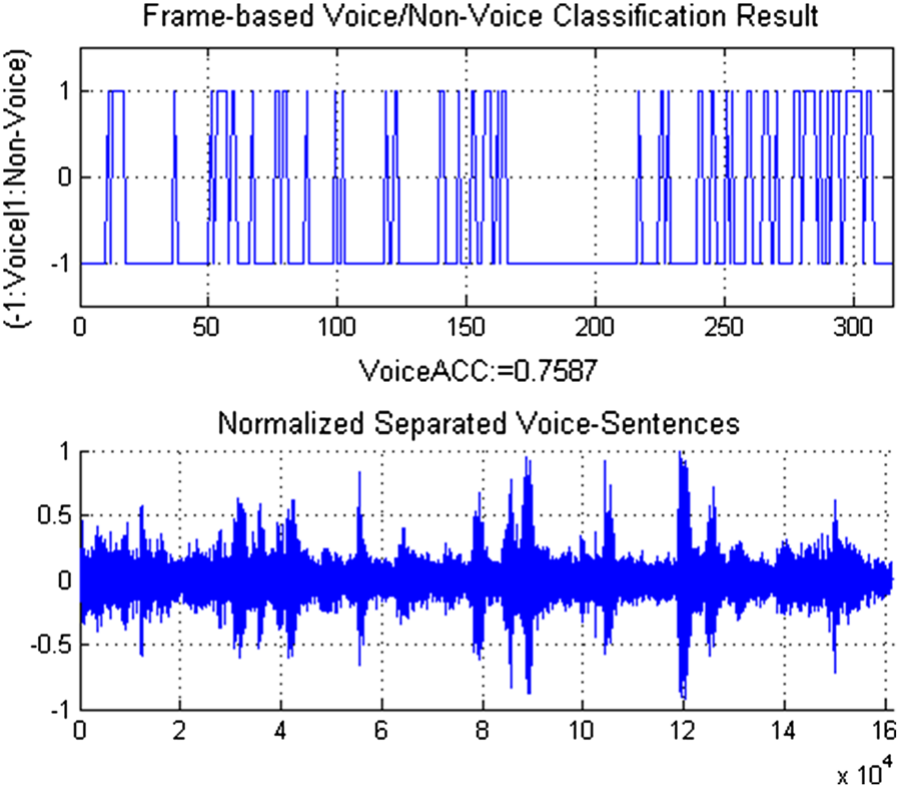
Voice/non-voice recognition of separated voice sentences

**Fig. 9:**
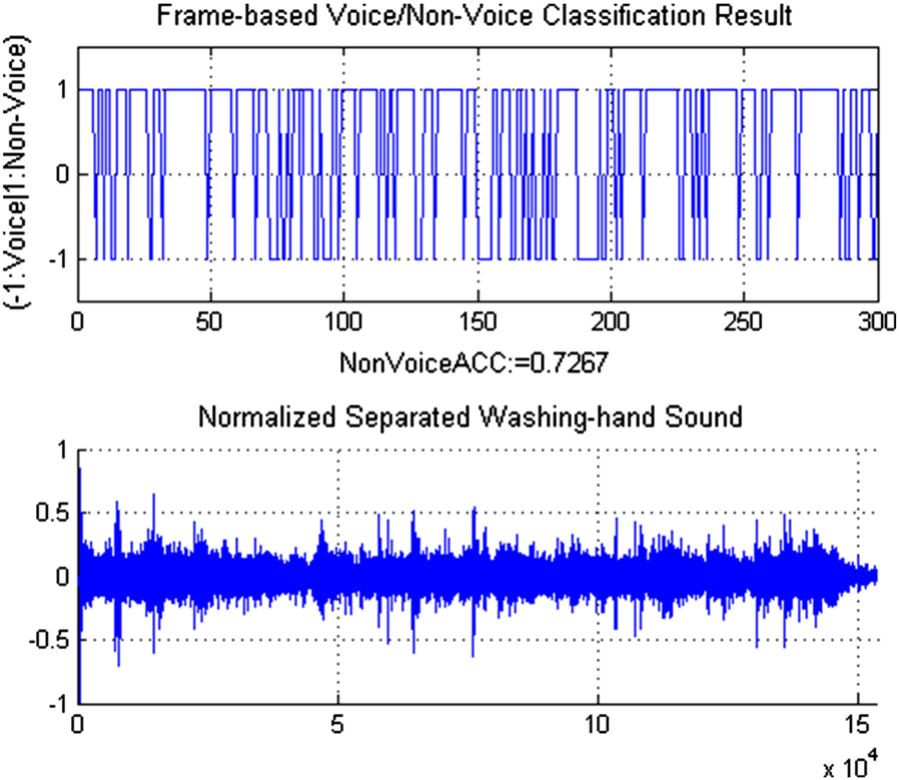
Voice/non-voice recognition of separated washing-hand sound

Event sounds and voice sounds in our SoundLib are randomly divided into training and testing data for the SVM-based VNR. We labelled non-voice for all frames in event sounds and voice for all frames in voice sounds. The VNR was trained by 36-MFCC feature vectors extracted from the audio segments with a length of 512 samples. The testing event sounds and voice sounds were divided into voice/non-voice pairs that were simultaneously played by two speakers at the SNR of 0 dB. The robot successful separated each pair into two different sounds. These separated sounds were used to test our SVM-based VNR algorithm. It shows that 95 % of these separated sounds have more than 70 % of frames that were recognized correctly into voice or non-voice frames. Therefore, when the thresholds of voice/non-voice decisions are set at 70 %, the voice/non-voice recognition results of the robot can reach an accuracy of 95 % for the whole separated sounds. As shown in Figs. 8 and 9, more than 75 % frames of the separated voice-sentence sound are voice, and more than 72 % frames of the separated washing-hand sound are non-voice.

**Fig. 10:**
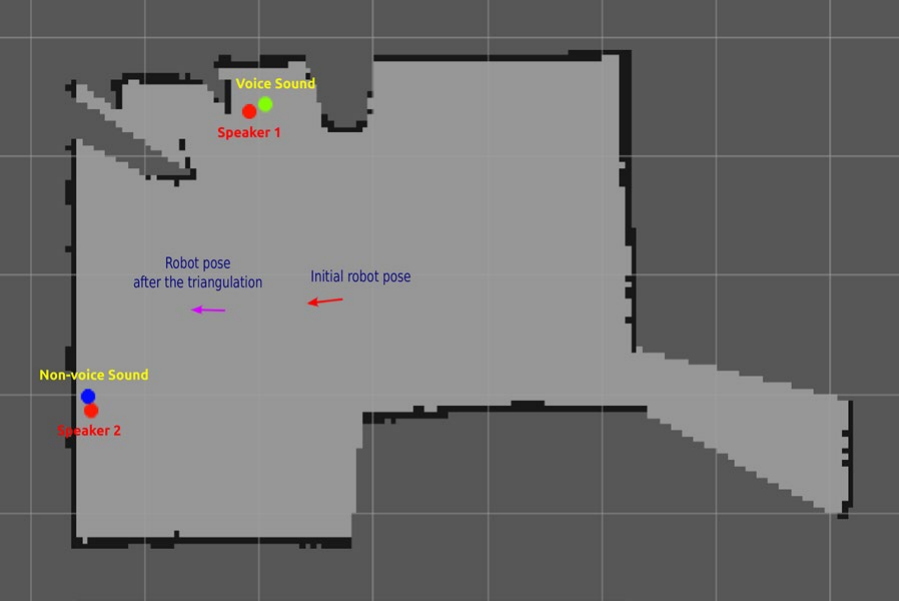
Sound position estimation by triangulation

**Fig. 11:**
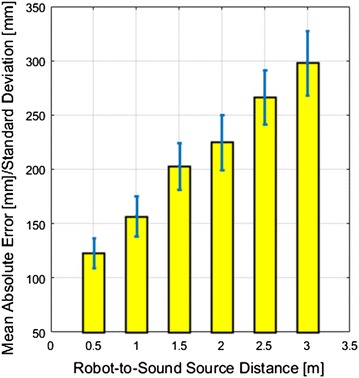
Results of sound position estimation by triangulation

### Sound source position estimation

Two speakers are deployed in the living room and the kitchen, respectively, and they simultaneously played voice sound and non-voice sound at a SNR of 0 dB. Figure 10 presents the results of sound positions estimated by the robot using triangulation. The ground truth positions of the two speakers are provided by the OptiTrack system and represented by the red dots in the 2D map created by the robot. The red arrow and the cyan arrow represent the robot poses in the 2D map at the beginning and the end of the triangulation, respectively. The estimated positions are represented in the map by the blue dot for the non-voice source and the green dot for the voice source. The mean absolute error and the standard deviation of estimated positions depend on the initial distance between the robot and the sound source as shown in Fig. 11.

**Fig. 12:**
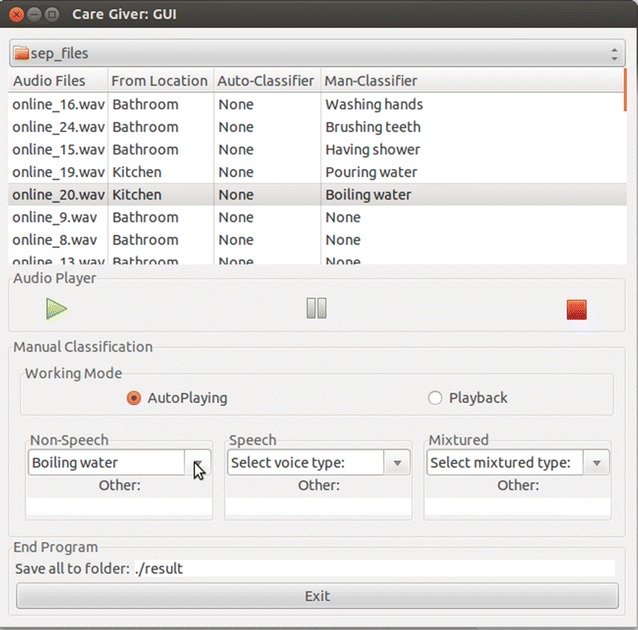
The user interface for human labelling of non-voice sounds

### Human-assisted sound classification

In our experiment, we were able to successfully assist the robot in labelling non-voice sounds when the caregiver is in another laboratory room. The GUI for sound labelling is shown in Fig. 12. Each of the separated non-voice sounds was sent to the caregiver in five-second segments that were saved into .wav files. They were also played on the GUI and the caregiver selected the appropriate labels by clicking on the combobox or inputting new labels. The labelling results were sent back to the robot using JSON format files that can be used in further training. Therefore, this application can be used for human–robot collaboration in detecting abnormal sounds in home environments.

**Fig. 13:**
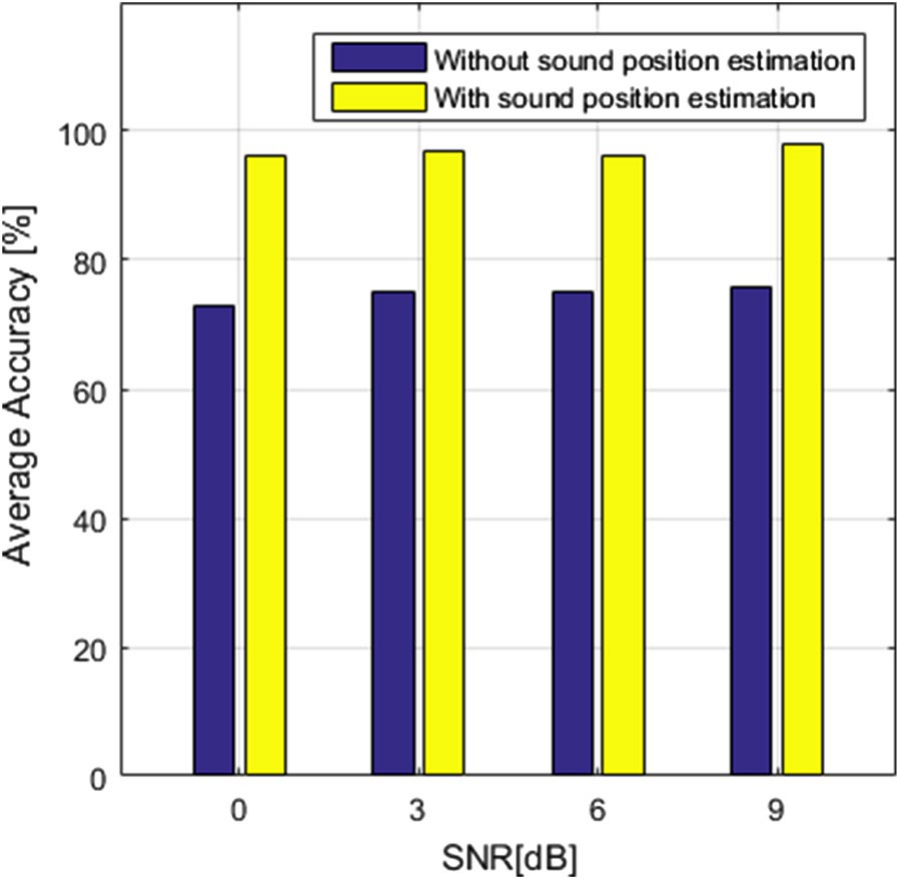
Average accuracy rates of human-assisted sound labelling

In order to evaluate human-assisted labelling, two speakers simultaneously played the voice sound and the event sound with the SNRs between the event sound and the voice sound at around 0, 3, 6, and 9 dB. In these cases, the voice sound is treated as the noise and its power is controlled by the audio player software on the audio node. The speaker that plays event sounds was moved around the testbed based on where the sounds should come from, for example boiling-water sound was played by the speaker in the kitchen. All 50 event sounds in the ASCC SoundLib were played. The robot estimates their position, separates, and recognizes them from the background of the voice sound, then sends separated event sounds to the remote caregiver for labelling. A total of 10 graduate students from our laboratory participated in this experiment as remote caregivers. The experiment consists of two different tests. In the first test, only the separated sounds were sent to caregivers. In the second test, both separated sounds and their position estimation were sent to caregivers. As shown in Fig. 13, the average accuracy rates of human-assisted labelling in the first test are around 75 %, but 98 % in the second test due to the position information is attached with each sound. Such contextual information provides reasonable hints for the caregivers to classify the sound events.

**Fig. 14:**
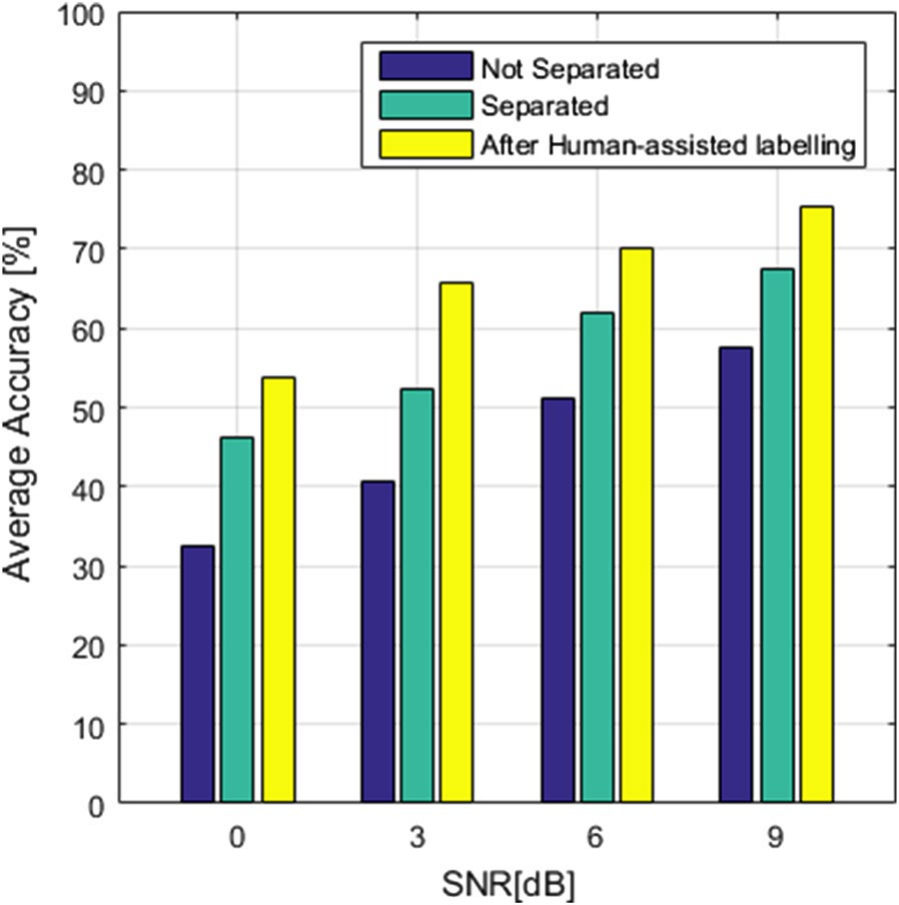
Average accuracy rates of sound event recognition with respect to different SNRs

Similarly, in order to evaluate autonomous sound event classification, two speakers simultaneously played the voice sound and the event sound. The SNRs between the event sound and the voice sound are approximately 0, 3, 6, and 9 dB. We tested the SVM-based SER with 20 classes of sound events (*boiling water, frying fries, making coffee, washing dishes, teapot whistle, filling water glass, washing hands, brushing teeth, soaping hands, having shower, washing machine, flushing toilet, drying hair, eating snack, glass dropping, door opening, yawning, coughing, laughing, others*). The average accuracy rates of sound event recognition with respect to SNRs are shown in Fig. 14. In the first experiment, clean event sounds in the ASCC SoundLib were used as the training data. The testing results without sound separation are very poor with average accuracy rates below 60 %. In the second experiment, the results are better when the sound separation is applied. These separated event sounds are labelled by the human and then used for training in the third experiment. The results are improved significantly.

## Conclusions

In this research, we proposed and developed human-assisted sound event recognition for home service robots. Besides implementing robot services based on ROS and the auditory services based on HARK, three functions were implemented for human-assisted sound event recognition: sound source position estimation based on triangulation, human-assisted sound event labelling, and autonomous sound event recognition. We tested and evaluated the above functions. Experimental evaluation verified that the remote caregiver and the robot can collaborate to facilitate sound event recognition while protecting human privacy. Overall, this system will help develop social intelligence for robot companions. The future work will develop algorithms for the robot to understand and predict the human activities and intentions through sound events.
